# Supportive care needs of patients following treatment for colorectal cancer: risk factors for unmet needs and the association between unmet needs and health-related quality of life—results from the ColoREctal Wellbeing (CREW) study

**DOI:** 10.1007/s11764-019-00805-6

**Published:** 2019-09-11

**Authors:** S. C. Sodergren, S. J. Wheelwright, N. V. Permyakova, M. Patel, L. Calman, P. W. F. Smith, A. Din, A. Richardson, D. Fenlon, J. Winter, J. Corner, C. Foster, Members of Study Advisory Committee, Jo Armes, Janis Baird, Andrew Bateman, Nick Beck, Graham Moon, Peter Hall, Karen Poole, Susan Restorick-Banks, Paul Roderick, Lesley Smith, Claire Taylor, Jocelyn Walters, Fran Williams

**Affiliations:** 1 Macmillan Survivorship Research Group, School of Health Sciences, Faculty of Environmental and Life Sciences, University of Southampton, Southampton, S017 1BJ UK; 2 Social Statistics and Demography, Social Sciences, University of Southampton, Southampton, UK; 3 University Hospital Southampton NHS Foundation Trust, Southampton, UK; 4 College of Human and Health Sciences, Swansea University, Swansea, UK; 5 Executive Office, University of Nottingham, Nottingham, UK

**Keywords:** Colorectal cancer, Supportive care needs, Health-related quality of life, Survivorship

## Abstract

**Purpose:**

To investigate unmet needs of patients with colorectal cancer (CRC) at the end of treatment and whether unmet needs improve over time. Identify predictors of need following treatment and whether unmet need is associated with worse health-related quality of life (HRQoL).

**Methods:**

As part of the UK ColoREctal Wellbeing (CREW) cohort study, patients treated for CRC completed the Supportive Care Needs Survey Short Form-34 (SCNS SF-34) 15 and 24 months following surgery, along with questionnaires measuring HRQoL, wellbeing, life events, social support, and confidence to manage their cancer before surgery, 3, 9, 15, and 24 months post-surgery.

**Results:**

The SCNS SF-34 was completed by 526 patients at 15 months and 510 patients at 24 months. About one-quarter of patients had at least one moderate or severe unmet need at both time points. Psychological and physical unmet needs were the most common and did not improve over time. Over 60% of patients who reported 5 or more moderate or severe unmet needs at 15 months experienced the same level of unmet need at 24 months. HRQoL at the beginning of treatment predicted unmet needs at the end of treatment. Unmet needs, specifically physical, psychological, and health system and information needs, were associated with poorer health and HRQoL at the end of treatment.

**Conclusions:**

Unmet needs persist over time and are associated with HRQoL. Evaluation of HRQoL at the start of treatment would help inform the identification of vulnerable patients. Assessment and care planning in response to unmet needs should be integrated into person-centred care.

**Implications for Cancer Survivors:**

Early identification of CRC patients at risk of unmet needs will help infrom personalised survivorship care plans. The implementation of personalised and tailored services are likely to confer HRQoL gains.

**Electronic supplementary material:**

The online version of this article (10.1007/s11764-019-00805-6) contains supplementary material, which is available to authorized users.

## Introduction

The experience of cancer and its treatment from the perspective of the patient is increasingly recognised as an integral part of health outcome assessment [1]. Patient-reported outcome measures (PROMs) focused on health-related quality of life (HRQoL) and satisfaction of care and needs, and collect information on physical or psychosocial problems that might otherwise be overlooked [2]. This information can be used to tailor treatment and care and has the potential to improve clinical outcomes [3–5]. Given that the physical and psychosocial impact of cancer and its treatment can be felt long after active treatment has been completed [6], it is important that patient-reported assessments continue beyond treatment.

The number of people living with and beyond cancer treatment is increasing and is predicted to reach 4 million in the UK by 2030 [7]. People living beyond colorectal cancer (CRC) form the largest group of survivors of cancer affecting men and women [8]. With improvements in early diagnosis and treatment, 76% of people in England diagnosed with CRC now survive a year and 57% 5 years [9]. The standard treatment pathway for CRC is surgery, with the addition of adjuvant chemotherapy for patients with Dukes’ C/high-risk Dukes’ B, neoadjuvant radio and/or chemotherapy for patients with rectal cancer, and neoadjuvant chemotherapy for patients with operable liver metastases. The rate of permanent stoma formation after rectal cancer surgery varies considerably and ranges from 9 to 50% across England [10].

A review of the difficulties encountered by people living 5 years post-cancer diagnosis found that 20–30% of survivors consistently reported problems associated with cancer and its treatment including physical problems, psychological distress, sexual problems, problems with relationships, and financial concerns [11]. Problems specifically affecting CRC survivors include the long-term physical and psychosocial effects of altered bowel function and stoma placement [12, 13]. Given finite resources, these problems might not be recognised or managed.

Unmet needs have been identified as one of the four main gaps in knowledge about the problems experienced by cancer survivors [14]. In addition, identification of survivors with the greatest needs may be important for developing appropriate and effective long-term survivorship care plans. Although there are several cancer-specific needs assessments designed for use with patients living with and beyond cancer [15–20], there is limited research into perceived unmet needs of patients, particularly within the early survivorship phase and change in needs over time [21, 22]. A review of patients’ needs assessment tools [23] identified the Supportive Care Needs Survey (SCNS) [16] as one of the most comprehensive instruments with respect to covering needs across the multi-dimensional aspects of health status as well as satisfaction. The SCNS assesses the perceived needs of people living with cancer, whether they remain unmet and the magnitude of such needs.

Armes et al. used the SCNS to investigate the supportive care needs of patients at the end of treatment for various cancer types including CRC and suggested that as many as half of cancer survivors might experience some form of unmet need, with psychological needs particularly flagged as inadequately met [21]. In addition, they found evidence for persistence in unmet needs over a 6-month period with no improvement in unmet needs for 60% of patients who had more than 5 unmet needs at the end of treatment [21]. Lam et al. also reported similar findings of prolonged unmet needs in CRC patients from the point of diagnosis to 12 months post-surgery [24].

Predictors of unmet need in cancer patients have been identified as age [25], active or advanced disease [16, 26], negative mood, other significant life events [20, 27–31], cancer-related rumination [24], educational attainment [24], low levels of social support (emotional or informational support and positive social interaction) [26], personality [32], low levels of physical activity [26], and maladaptive coping strategies such as anxious preoccupation, hopeless/helplessness, and avoidance [26]. However, previous studies only considered a small number of potential predictors.

This limitation was addressed by the UK ColoREctal Wellbeing study (CREW), a large-scale prospective cohort study investigating factors associated with recovery of health and wellbeing following a diagnosis of CRC [33]. The CREW domains of assessment were informed by a conceptual framework of recovery following cancer diagnosis and treatment [34] which hypothesises that a number of factors, including the supportive care needs of patients, influence the course of recovery. CREW used the SCNS Short Form 34 (SF-34) [16] to measure patient-perceived unmet needs at 15 months and 24 months following surgery, at which time any adjuvant treatment would have ended. The rationale for selecting these time points was that needs might be heightened during the transition period between active treatment and the recovery phase as clinician involvement in care declines and patients find themselves having to self-manage their condition.

The main aim of this paper is to describe patient-reported unmet needs after treatment for CRC and 9 months later. In particular, to investigate any change in perceived needs over time and to predict which individuals are more likely to have unmet needs at the end of treatment. A secondary aim was to investigate associations between unmet need following treatment and HRQoL outcomes, with the hypothesis that high unmet needs are associated with poorer recovery of health and wellbeing.

## Method

### Study design and participants

CREW is a multicentre, prospective cohort study of newly diagnosed patients with CRC, treated with curative intent in 29 cancer centres across the UK. Patients were eligible if they (a) had a diagnosis of non-metastatic CRC (Dukes’ A–C), (b) were awaiting primary surgery with curative intent (patients who had been identified as eligible and admitted for emergency surgery were also included), (c) ≥ 18 years old, and (d) had the ability to complete questionnaires. Recruitment took place between November 2010 and March 2012; the aim was to include every eligible patient diagnosed at each cancer centre during the centre’s recruitment period. Further details relating to eligibility, recruitment strategy, and sample size are provided elsewhere [33].

Written consent was obtained and baseline questionnaires completed prior to surgery whenever possible. Socio-demographic information including gender, age, ethnicity, employment, and domestic status was also collected at consent. Clinical details including tumour site (colon, rectum), Dukes’ stage (A, B, C1, and C2), and treatment (neoadjuvant, adjuvant, and stoma formation) were extracted from medical records at 6 months and verified at 24 months post-surgery. Self-reported comorbidities were recorded from 3 months post-surgery.

Patient self-report questionnaires also measured a broad range of issues including supportive care needs, life events (e.g. spousal/relational problems, bereavement, financial problems), health status, HRQoL, wellbeing, social support, and self-efficacy. Follow-up mailed questionnaires were completed at 3, 9, 15, and 24 months post-surgery (longer term data collection continued for up to 5 years).

The study was approved by the UK National Health Service National Research Ethics Service (REC reference number: 10/H0605/31).

### Measures

Full details of the repeated measures used in the CREW study are provided elsewhere [33].

#### Unmet needs

The SCNS SF-34 [16] was used to assess perceived needs and whether they had been met at 15- and 24-month follow-up points. The scale includes 34 items covering five domains of need: physical/daily living, psychological, sexuality, patient care and support, and health system and information. Each need is rated on a 5-point Likert-type scale with responses 1 (“Not applicable” or “No need”) and 2 (“Need satisfied”) indicating no outstanding need while 3, 4, and 5 measure degree of unmet need ranging from “Low” (little need for additional help) to “High” (strong need for additional help).

### Life events

The incidence of significant life events in the past 6 months was recorded from 3-month follow-up using a modified version of the list of threatening life experiences [35], with death of a spouse and child presented as separate life events and death of a pet and moving house added.

### Health status, HRQoL, and wellbeing

The EuroQol 3-level version (EQ-5D-3L) [36] measures generic health status across five domains: mobility, self-care, usual activities, pain/discomfort, and anxiety/depression each scored as none/some/severe problems.

The Quality of Life of Adult Cancer Survivors (QLACS) Scale Part 1 [37] measures 28 QoL issues across eight generic domains which are not necessarily attributable to cancer: negative feelings, positive feelings, cognitive problems, pain, sexual interest and sexual function (merged together), energy/fatigue, and social avoidance.

The European Organisation for Research and Treatment of Cancer (EORTC) 30-item Core measure (QLQ-C30) [38] and the CRC module (QLQ-CR29) [39] were included from 3 months onwards. The QLQ-C30 includes five functional scales, three symptom scales, six single items, and a Global health status/QoL scale. The QLQ-CR29 supplements the QLQ-C30 with 29 colorectal cancer-specific items, from which are derived four functional subscales (body image, anxiety, weight, and sexual interest) and 19 single item scales. A linear transformation is applied to produce subscale scores with a possible range from 0 to 100, with high scores indicating better functioning on the functional and Global health/QoL scales but poorer symptoms on the symptom scales/items.

The Personal Wellbeing Index–Adult (PWI-A) [40] includes eight items of satisfaction corresponding to standard of living, health, achieving in life, relationships, safety, community connectedness, future security, and spirituality/religion.

Anxiety and depression were assessed using the State-Trait Anxiety Inventory (STAI) [41] and the Centre for Epidemiologic Studies Depression Scale (CES-D) [42].

Positive and negative mood states were measured using 10 questions (5 questions for each mood state) from the Positive and Negative Affect Scale (PANAS) [43].

### Social support

The MOS Social Support Survey (MOS-SSS) [44] provides an indication of how often different types of support are perceived to be available including emotional/informational, tangible, affectionate, and positive social interaction.

### Self-efficacy

The Self-efficacy for Managing Chronic Disease Scale (Lorig) [45] includes six items measuring confidence in different areas.

### Analysis

Univariate analysis of baseline characteristics of participants was conducted using a chi-squared test to compare those who completed at least one SCNS domain at 15 months with those who did not. A multivariable logistic regression model was additionally constructed to assess the differences once all significant characteristics were mutually adjusted for (*p* < 0.05).

Independent variables were divided into five thematic blocks: (1) socio-demographic characteristics (gender, age, employment, and domestic status), (2) clinical details (tumour site, staging, treatment, stoma, co-morbidities), (3) psychosocial measures (EQ-5D, QLACS-GSS, PWI-A, CES-D, STAI, PANAS, MOS, Lorig), (4) cancer HRQoL (QLQ-C30), and (5) CRC-specific HRQoL (QLQ-CR29). Due to a skewed distribution in most of the psychosocial measures and EORTC subscales, all scores, with the exception of QLACS-GSS and the QLQ-C30 Global health/QoL score, were converted into categorical covariates. See Supplementary Material 1: Table 1.2, footnote 1 for details.

Descriptive measures were used to examine the prevalence of total unmet supportive care needs. Consistent with previous research on unmet needs [21], the number of participants with no, few (1–4), and many (5 or above) moderate or severe unmet needs was calculated. In terms of missing data, patients were identified as having 5 or more unmet needs even if some of the composite questions were missing (i.e. at least 5 were rated as severe or moderate), otherwise the data were excluded from the analysis. To investigate change in perceived needs over time, a chi-squared test was used to test for differences (marginal homogeneity) in the distribution of both overall and SCNS domain unmet needs between the two time points of 15 and 24 months. In addition, the ten most common moderate/severe unmet needs at 15 and 24 months post-surgery were ranked and compared.

To predict which individuals are more likely to have unmet needs at the end of treatment (15 months post-surgery), logistic regression models were used to assess the baseline predictors of unmet need for each SCNS domain. In line with previous research using the SCNS [16, 21], each SCNS domain of unmet needs was dichotomised into no or low unmet need (score = 1 to 3) and moderate or severe unmet need (score = 4 or 5). Independent variables were taken at the earliest time point available which was at baseline except for the comorbidity status and the EORTC measures (QLQ-C30 and QLQ-CR29), which were first completed at 3 months. Therefore, we restricted our sample to those respondents who participated at three time points: baseline, 3 months, and 15 months. For each SCNS domain, the final regression models were adjusted for age as an important demographic effect-modifier.

To investigate the associations between the SCNS domains of unmet need following treatment (15 months) and HRQoL outcome (also at 15 months), an ordinary least squares (linear) regression model was produced. The QLQ-C30 Global health/QoL scale score at 15 months was preferred as the (continuous) outcome measure since there was no evidence against the assumption that the resulting residuals for the linear regression model were normally distributed. All five SCNS domains (physical/daily living, psychological, sexuality, patient care and support, and health system and information) were the covariates of interest at 15 months and entered into the model together. The model was adjusted for the socio-demographic and clinical characteristics (Blocks 1–2, including disease recurrence and negative life events). Blocks 3–5 were not included because of the hypothesised interaction between these variables and supportive care needs. Additional regression models were produced to explore the association between each individual SCNS domain and HRQoL outcome at 15 months, controlling for the significant confounders.

Random clustering effects of cancer centre were assessed by fitting centre separately as a fixed variable to each regression model and as a random effect. Both effects were negligible for all models; therefore, centre was not included in subsequent models.

For all regression models, a forward stepwise selection approach was used, which was applied in two steps: first, separately to each thematic block of independent variables; second, to the final set of those covariates which remained significant in each block.

The significance level was fixed at 5% and analyses were carried out using Stata Corp. StataSE 14 and IBM SPSS Statistics 24.

## Results

### Characteristics of participants

Full details of the characteristics of the sample are provided elsewhere [46].

At 15 months follow-up, 526 patients (70% of those who gave a full consent at baseline) completed the SCNS as part of their assessment. At 24 months follow-up, 510 (67% of baseline completers) patients completed the SCNS with 448 (59% of baseline completers) completing the measure at both time points.

Completers and non-completers of the 15-month SCNS were broadly similar in terms of baseline socio-demographic and clinical characteristics (Supplementary Material 1: Table 1.1), although non-completers were significantly older and were more likely to be widowed or single (*p* < 0.001). No significant differences were found in the clinical characteristics of the participants by their status of SCNS completion at 15 months (*p* > 0.05). Difference in psychosocial characteristics and EORTC subscales (Supplementary Material 1: Table 1.2) disappeared once age and domestic status of respondents were controlled for in the multivariable model (Supplementary Material 1: Table 1.3).

### Unmet needs following treatment

**Fig. 1:**
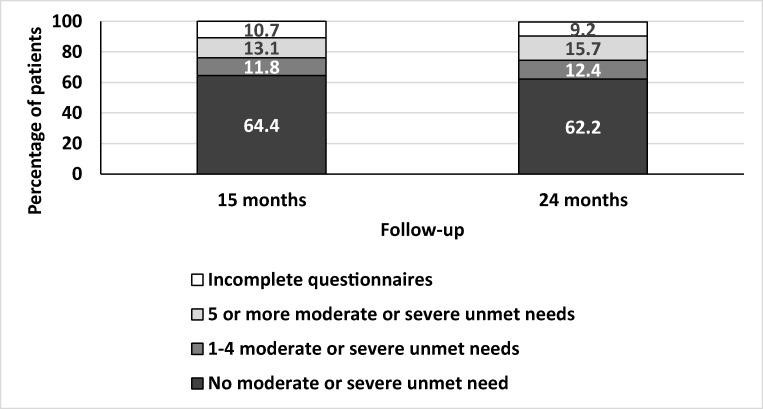
Moderate or severe unmet needs at 15 months and 24 months post-surgery

The prevalence of unmet need at 15 months and 24 months post-surgery for each of the 34 items in the SCNS is available as supplementary material (see Supplementary Material 2). At 15 months following surgery, 24.9% of participants reported at least one moderate or severe unmet need (score of 4 or 5), an additional 18.4% reported at least one low unmet need (score of 3) and 46% patients reported no moderate or severe unmet needs (scores of 1 or 2). Incomplete questionnaires meant that 10.7% of participants could not be included in this analysis. Unmet needs prevalence was similar at 24 months with 28.1% reporting at least one moderate or severe unmet need. Figure 1 shows the prevalence of moderate or severe needs at each time point, in terms of those with many unmet needs (> 5) and those with fewer unmet needs (< 4).

The prevalence of unmet needs according to the area of need is shown in Table 1, with the most common moderate or severe unmet needs across the two time points falling within the psychological domain. In terms of item rankings shown in Table 2, fear of cancer spreading, restricted activities, and lack of energy or tiredness were the most prominent unmet needs at both time points.

**Table 1:** Number (percentage) participants at 15 months and 24 months expressing at least one moderate or severe unmet need across the SCNS domains

Domain	15 months SCNS (526 completed)		24 months SCNS (510 completed)		Chi-squared test
	At least one moderate/high unmet need	Missing data on all needs for the domain	At least one moderate/high unmet need	Missing data on all needs for the domain	
	*N* (%)	*N* (%)	*N* (%)	*N* (%)	*p* value
Psychological	90 (17.1)	6 (1.1)	106 (20.8)	11 (2.2)	0.060
Physical and daily living	83 (15.8)	4 (0.8)	86 (16.9)	10 (2.0)	0.229
Sexuality	45 (8.6)	10 (1.9)	42 (8.2)	15 (2.9)	1.000
Patient care and support	40 (7.6)	3 (0.6)	43 (8.4)	5 (1.0)	0.618
Health system and information needs	58 (11.0)	6 (1.1)	66 (12.9)	14 (2.7)	0.193

**Table 2:** Top 10 most prevalent moderate or severe unmet needs at 15 and 24 months following surgery

Time point	SCNS item	Rank	Moderate/severe unmet need	Missing
			*N* (%)	*N* (%)
15 months *N* = 526	Not being able to do the things you used to do	1	59 (11.2)	8 (1.5)
	Fears about the cancer spreading	2	50 (9.5)	16 (3.0)
	Lack of energy/tiredness	3	48 (9.1)	14 (2.7)
	Uncertainty about future	4	46 (8.7)	14 (2.7)
	Concerns about the worries of those close to you	5	44 (8.4)	14 (2.7)
	Changes in sexual feelings	6	39 (7.4)	22 (4.2)
	Changes in sexual relationships	7	35 (6.7)	21 (4.0)
	Worry the results of treatment are beyond your control	8	34 (6.5)	16 (3.0)
	Work around home	9	32 (6.1)	20 (3.8)
	Anxiety	10	31 (5.9)	19 (3.6)
24 months *N* = 510	Fears about the cancer spreading	1	56 (11.0)	19 (3.7)
	Not being able to do the things you used to do	2	54 (10.6)	17 (3.3)
	Lack of energy/tiredness	3	52 (10.2)	22 (4.3)
	Anxiety	4	51 (10.0)	20 (3.9)
	Feeling down or depressed	5	50 (9.8)	15 (2.9)
	Concerns about the worries of those close to you	6	49 (9.6)	15 (2.9)
	Feelings of sadness	7	48 (9.4)	19 (3.7)
	Uncertainty about future	8	43 (8.4)	16 (3.1)
	Worry the results of treatment are beyond your control	9	40 (7.8)	15 (2.9)
	Pain	10	36 (7.1)	21 (4.1)

### Change in needs over time

Table 3 reports change in total unmet needs from 15 to 24 months. Of the 327 patients with no needs at 15 months, 49 (15%) went on to report at least one need at 24 months. Of the patients who completed the SCNS at both time points, 54 patients reported having five or more moderate or severe unmet needs at 15 months and 35 of these (63.6%) also reported five or more unmet needs at 24 months. However, the chi-squared test revealed no significant change in overall needs between the two time points (*p* value < 0.05) and this was also true for the change in SCNS domains over time (*p* values presented in Table 1).

**Table 3:** Change in frequency of total unmet needs from 15 to 24 months

15 months	24 months							
	No needs		1–4 needs		5+ needs		Total	
	*N*	%	*N*	%	*N*	%	*N*	%
No needs	278	62.1	32	7.1	17	3.8	327	73.0
1–4 needs	29	6.5	26	5.8	12	2.7	67	15.0
5+ needs	13	2.9	6	1.3	35	7.8	54	12.1
Total	320	71.4	64	14.3	64	14.3	448	100

Excludes 24 months non-completers (chi-squared test *p* = 0.352)

### Baseline predictors of supportive care needs after treatment

Since there was no significant change in unmet needs between 15 and 24 months, we selected the 15-month follow-up assessment to model predictors of unmet need. The within-blocks logistic regression models are available in Supplementary Material 3. Table 4 presents the final multivariable logistic regression models separately for each of five SCNS domains.

**Table 4:** Multivariable logistic regression models of at least one moderate or severe unmet need for each SCNS domain at 15 months, baseline covariates

Independent (baseline) variables	OR	(OR 95% CI)
Model 1: high physical and daily living needs		
Unemployed/retired (ref: employed)	3.82**	(1.43; 10.23)
At least one comorbidity (ref: none)	2.74*	(1.12; 6.71)
QLQ-C30 role functioning problem (ref: no problem)	2.66**	(1.33; 5.31)
QLQ-CR29 fatigue problem (ref: no problem)	4.17***	(1.98; 8.81)
QLQ-CR29 buttock pain problem (ref: no problem)	4.24***	(1.88; 9.54)
QLQ-CR29 body image problem (ref: no problem)	2.29*	(1.17; 4.48)
Model 2: high psychological needs		
Adjuvant therapy (ref: no)	3.74***	(1.98; 7.10)
QLACS-GSS score	1.02***	(1.01; 1.03)
QLQ-C30 role functioning problem (ref: no problem)	3.17***	(1.72; 5.82)
QLQ-CR29 buttock pain problem (ref: no problem)	3.57**	(1.62; 7.87)
QLQ-CR29 body image problem (ref: no problem)	2.31*	(1.21; 4.42)
Model 3: high sexuality needs		
Women (ref: men)	0.18**	(0.07; 0.51)
Low/moderate self-efficacy (Lorig) (ref: (very) confident)	2.32*	(1.03; 5.21)
QLQ-CR29 buttock pain problem (ref: no problem)	3.11*	(1.22; 7.91)
QLQ-CR29 body image problem (ref: no problem)	3.59**	(1.60; 8.07)
QLQ-CR29 dysuria problem (ref: no problem)	10.04*	(1.46; 69.02)
Model 4: high patient care and support needs		
QLQ-C30 Global health/quality of life score	0.97**	(0.95; 0.99)
QLQ-CR29 body image problem (ref: no problem)	5.44***	(2.33; 12.69)
QLQ-CR29 blood or mucus in stool (ref: no problem)	7.50**	(2.03; 27.74)
Model 5: high health system and information needs		
Adjuvant therapy (ref: no)	2.17*	(1.14; 4.16)
QLQ-C30 blood or mucus in stool (ref: no problem)	7.41**	(2.13; 25.72)
QLQ-CR29 body image problem (ref: no problem)	5.27***	(2.74; 10.12)

Each model is adjusted for age of respondents

*p < 0.05; **p < 0.01; ***p < 0.001

*Abbreviations*: *QLQ-C30*, EORTC QLQ-C30; *QLQ-CR29*, EORTC QLQ-CR29; *QLACS-GSS*, Quality of Life in Adult Cancer Survivors Generic Summary Score; *Lorig*, The Self-efficacy for Managing Chronic Disease Scale

While several socio-demographic, clinical, and psychosocial characteristics place patients at greater risk of individual unmet needs, HRQoL, as assessed by the QLQ-C30 or QLQ-CR29, was predictive of unmet needs across all SCNS domains. In particular, problems with body image (QLQ-CR29 subscale) at baseline were predictive of all unmet needs domains at 15 months. In addition, QLQ-CR29 buttock pain was significantly associated with physical, psychological, and sexuality unmet needs with an odds ratio varying between 3.1 and 4.2 for the three SCNS domains. At 15 months, unmet sexuality needs were most prominent among patients with dysuria problems (QLQ-CR29) at baseline. Patients with blood or mucus in the stools (QLQ-CR29) at baseline had on average 7 times higher odds of having unmet patient care and support needs and health system and information needs. Finally, patients with reduced role functioning (QLQ-C30 subscale) had 2–3 times higher odds of having high unmet physical and psychological needs. With the exception the measure of self-efficacy (Lorig), none of the psychosocial measures (EQ-5D, QLACS-GSS, PWI-A, CES-D, STAI, PANAS, MOS) were predictive of unmet need at the end of treatment,

### Association between Global health/QoL and supportive care needs at 15 months

Supplementary Material 4 shows the variables from each thematic block, which were significantly associated with Global health/QOL (QLQ-C30) score at 15 months. When separate regression models with each SCNS domain (as the covariates of interest at 15 months) were generated, all domains were significantly associated with Global health/QOL. Independently from each other, each domain remained significant after adjusting for the confounders from Blocks 1–3 (see the models in Supplementary Material 5). However, when the five SCNS domains were entered into one model together, even without the confounders, only three domains remained statistically significant (physical and daily living needs, psychological needs, and health system and information needs, see Model 4 in Supplementary Material 4).

The final multivariable model (model 4 adjusted for the significant confounders) is shown in Table 5. Physical and daily living needs had the largest association with Global health/QoL: Someone who experienced at least one severe or moderate unmet physical and daily living need had on average a 15.1-point (CI [− 19.45; − 10.79]) decreased Global health/QoL score compared with someone without an unmet need within this SCNS domain. Unmet psychological need and health system and information also had large associations with Global health/QoL.

**Table 5:** Multivariable linear regression model of QLQ-C30 Global health/QoL at 15 months, adjusted for the significant covariates from each thematic block^1^

Independent (at 15 months) variables	Coefficient	(95% CI)
Physical and daily living needs (ref: no need/low level)	0.00	
Yes, high level of this domain of needs	− 15.12***	(− 19.45; − 10.79)
Psychological needs (ref: no need/low level)	0.00	
Yes, high level of this domain of needs	− 7.33***	(− 11.61; − 3.06)
Health system and information needs (ref: no need/low level)	0.00	
Yes, high level of this domain of needs	− 6.25**	(− 10.77; − 1.74)
Domestic status (ref: married/cohabiting)	0.00	
Single/never married/divorced/widowed	− 4.31**	(− 7.28; − 1.34)
Comorbidities (ref: none)	0.00	
Yes, at least one	− 7.43***	(− 10.56; − 4.30)
Neoadjuvant therapy (ref: none)	0.00	
Yes, any (chemotherapy/radiotherapy /both)	− 4.23**	(− 7.68; −0.79)
Had any negative life event in the last 6 months (ref: none)	0.00	
Yes, at least one	− 3.97**	(− 6.89; − 1.05)

The model is adjusted for age of respondents; *p < 0.05; **p < 0.01; ***p < 0.001

^1^See Supplementary material 4 for the significant covariates from each thematic block

### Sensitivity analysis

Although the distribution of residuals in the linear regression model for QLQ-C30 Global health/QoL met the normality assumption, we additionally tested whether outliers biased the association between the outcome and the SCNS domains. The regression model excluding seven outliers provided similar results for the SCNS domains (see Supplementary Material 6: Table 6.1, Model 1).

## Discussion

This study found that 43% of people who have undergone surgery for CRC still have unmet needs 15 months later (moderate or severe for 25%) and these needs persist at 24 months. Poor HRQoL, measured shortly after diagnosis, was predictive of unmet needs following treatment thus the capture of HRQoL data, as part of a comprehensive assessment around the time of diagnosis, would help identify those at risk of unmet needs. Furthermore, after treatment had finished, having an unmet need, particularly with respect to physical or daily living aspects, psychological and health system and information was associated with poorer global HRQoL and health status.

Compared with previous research using the SCNS SF-34 with mixed cancer cohorts [16, 21, 22, 26], our sample reported fewer unmet needs. In a study by McDowell et al. [22], unmet needs were reported by approximately two-thirds of cancer patients (with mixed tumour types) at the end of treatment, and half at 6 months post-treatment. Patients in our study also presented with fewer needs compared with CRC patients in other studies, for example Jorgensen et al. [25] found that unmet needs at 3 months post-surgery were reported by 79% of patients aged < 65 years and 65% of patients aged 65 years and older. However, the previous studies included patients with more advanced disease (including those with metastases—Dukes’ stage D). Our method for calculating percentages for unmet need prevalence is also more conservative than other methods, given that we based it on the total number in the sample, rather than just those expressing a need. In addition, we may have underestimated the level of unmet need because the non-completers in our study had greater morbidity at baseline and thus may have had the greatest need. However, the level of attrition in our study between baseline and follow-up assessments was low.

Despite the relatively low prevalence of moderate and severe unmet need in our sample, we identified a group of patients with a high number (> 5) of moderate or severe unmet needs: 13% of patients at the end of treatment and 16% 9 months later had at least five moderate or severe unmet needs. It is also worth noting that there was a small proportion (15%) of patients who reported no moderate or severe unmet need at the end of treatment and went on to report one or more moderate or severe unmet needs at 24 months.

The significant physical and psychological burden imposed by cancer and its treatment is reflected in the prevalence of unmet need within the physical and daily living needs and psychological needs domains, and concurs with previous findings [21, 26]. In line with previous findings [26, 47], we found that patients’ worries extend beyond their own health and future to those of other people close to them, which was identified as the fifth and sixth most prevalent unmet need for the two respective time points.

In our study, unmet needs were assessed at a time when treatment had ended and patients were likely to be less closely monitored and have fewer opportunities to discuss and address their concerns with healthcare professionals. Even when patients have access to health professionals, evidence suggests that psychological needs of patients with cancer are often overlooked, and patients may perceive that their psychological needs are unmet. Research by DiFabio et al. [48] compared surgeons’ and patients’ evaluations of areas of need following CRC resection. Their findings suggest that while 26% of patients identified addressing emotional problems as important, these were not recognised as a need for attention by any of the surgeons surveyed. In addition, health professionals might not feel adequately equipped or have the time to handle psychological concerns including fear of recurrence [49]. Thus, healthcare professionals might avoid asking about concerns which they cannot address.

Pre-treatment characteristics were predictive of unmet needs reported 15 months later. Notably, there was evidence to suggest treatment type had the greatest impact on unmet psychological needs, with patients who received adjuvant treatment more likely to report unmet moderate or severe psychological needs compared with those receiving no adjuvant treatment. This can be explained by the greater demands placed on patients and potential psychological effects of undergoing a combined treatment regimen. We also found that patients with a diagnosis of at least one other illness in addition to cancer had more physical unmet needs which is consistent with previous findings [50] and suggests that other diagnoses are likely to bring more physical demands.

Our findings also suggest that patients not in employment or retired were more likely to report unmet physical and daily living needs. Similar findings have been previously noted by Boyes et al. [26], where patients with more physical and daily living needs were unable to work or were older and retired. We also found that patients who were not confident in managing their illness were more likely to report unmet sexual needs. Low self-efficacy has previously been identified as predictive of poor QoL in this study sample [51].

To our knowledge, this study is the first to explore the broad spectrum of HRQoL (symptoms and functioning) soon after diagnosis as predictors of future unmet needs, with previous research often cross-sectional in nature and limited to socio-demographic and clinical correlates of unmet needs [25]. HRQoL scores predicted all 5 domains of unmet need at 15 months, with poorer HRQoL scores on the symptom scales increasing the odds of unmet need. In particular, patients with poorer body image were more likely to have unmet needs across all domains. Impaired body image is common in CRC and linked to potential stoma formation and changes in bodily function following CRC surgery [52]. Studies evaluating the impact of colorectal cancer on body image tend to focus on psychosocial functioning and sexuality concerns [53]. Our findings suggest that body image concerns have widespread consequences for unmet needs and underscore the importance of attending to body image especially as the greatest impact was on patient care and support needs.

Our findings also suggest that unresolved symptoms and functioning issues can cause difficulties that can remain unaddressed. For example, patients with higher scores in terms of buttock pain had more physical, psychological, and sexuality unmet needs. Dysuria was the greatest predictor of unmet sexuality needs while problems with blood in mucus or stools placed patients at greater risk of unmet patient care and support and healthcare and information unmet needs.

Previous research has examined the association between unmet needs and HRQoL and suggests unmet needs are a more significant correlate of HRQoL in cancer patients than socio-demographic or clinical characteristics [54, 55]. In addition, Husson et al. found that addressing unmet need leads to improved HRQoL [56]. Our study confirms previous research and suggests that moderate or severe unmet needs result in a decreased Global health/QoL score of up to 15 points. Unmet physical needs had the largest association with Global health/QoL, suggesting that the assessment and treatment of physical and daily living needs is particularly important. Psychological, and health system and information needs were also significantly associated with reduced health and wellbeing, suggesting that these are also important areas for intervention.

### Limitations

Our study includes a highly representative sample of the eligible CRC patients from the recruitment period, with high rates of retention. However, comparison of the participants who remained in the study at 15 months with baseline participants suggests the possibility of underestimation of unmet need at 15 months given that those who left the study by this point might be at risk of greater unmet needs.

Although the study included a large number of potential factors associated with unmet need and HRQoL, some factors were under-represented in the analyses because of low prevalence or high rates of missing data. Other potential key variables such as specific coping styles and level of physical activity which have previously been explored and identified as playing a significant role [26], were not included in the analyses.

It would have been valuable to carry out a needs assessment at diagnosis as has been done in a previous study [24]. However, we could determine whether patients had ever had the need, whether the need was satisfied or whether there is an outstanding need for support. For the majority of patients, needs were identified as not applicable suggesting they had never required help. In addition, extending the timeframe of SCNS SF-34 assessment points following treatment might have provided greater scope for a reduction of unmet needs. For example, it is possible that sexuality needs are not addressed until later on in the illness continuum. Future research should continue to measure supportive care needs over a more prolonged period of time.

Finally, while we measured supportive care needs using a well-validated instrument (SCNS SF-34), it is possible that certain needs might have been overlooked. Thereby, the SCNS SF-34 may not fully capture the unique needs of patients with CRC in the early phase following treatment.

## Conclusion

About a quarter of our sample of patients with CRC reported persistent moderate or severe unmet needs at the end of treatment, particularly related to psychological and physical concerns. Supportive care services should target those at risk of prolonged high unmet needs, and the implementation of personalised and tailored services are likely to confer HRQoL gains. Our findings suggest that identification of vulnerable patients should look beyond socio-demographic and clinical parameters and include an evaluation of patients’ HRQoL at the beginning of treatment. Assessment and care planning in response to unmet needs should be integrated into personalised survivorship care.

## Electronic supplementary material

ESM 1 (DOCX 38 kb)

ESM 2 (DOCX 23 kb)

ESM 3 (DOCX 17 kb)

ESM 4 (DOCX 14 kb)

ESM 5 (DOCX 13 kb)

ESM 6 (DOCX 15 kb)
