# Supplementary material for: Supportive care needs of patients following treatment for colorectal cancer: risk factors for unmet needs and the association between unmet needs and health-related quality of life—results from the ColoREctal Wellbeing (CREW) study
Source: J Cancer Surviv. 2019 Sep 11;13(6):899–909. doi: 10.1007/s11764-019-00805-6 (PMC6881415; doi:10.1007/s11764-019-00805-6)
Supplement: Supplementary file 1 — (DOCX 38 kb) [file 11764_2019_805_MOESM1_ESM.docx]

**Supplementary Material 1**

**Table 1.1**: Baseline socio-demographic and clinical characteristics of participants according to whether
or not they completed the SCNS at 15 months

| Independent variables | Non-completers (N=250)* | | Completers (N=506)* | | P-value1 |
| --- | --- | --- | --- | --- | --- |
|  | N | % | N | % |  |
| Gender |  |  |  |  | 0.333 |
| Male | 141 | 56.4 | 304 | 60.1 |  |
| Female | 109 | 43.6 | 202 | 39.9 |  |
| Age |  |  |  |  | < 0.001 |
| <50 | 17 | 6.8 | 30 | 5.9 |  |
| 51-60 | 31 | 12.4 | 82 | 16.2 |  |
| 61-70 | 74 | 29.6 | 216 | 42.7 |  |
| 71-80 | 79 | 31.6 | 144 | 28.5 |  |
| >80 | 47 | 18.8 | 34 | 6.7 |  |
| missing | 2 | 0.8 | 0 | 0.0 |  |
| Employment status |  |  |  |  | 0.050 |
| Employed | 53 | 21.2 | 149 | 29.4 |  |
| Unemployed | 13 | 5.2 | 21 | 4.2 |  |
| Retired | 182 | 72.8 | 331 | 65.4 |  |
| Unknown | 2 | 0.8 | 5 | 1.0 |  |
| Domestic status |  |  |  |  | < 0.001 |
| Married | 143 | 57.2 | 352 | 69.6 |  |
| Living with a partner | 17 | 6.8 | 22 | 4.3 |  |
| Widowed | 53 | 21.2 | 57 | 11.3 |  |
| Divorced / Separated | 16 | 6.4 | 48 | 9.5 |  |
| Single | 19 | 7.6 | 25 | 4.9 |  |
| Unknown | 2 | 0.8 | 2 | 0.4 |  |
| Tumour site |  |  |  |  | 0.147 |
| Colon | 169 | 67.6 | 317 | 62.6 |  |
| Rectum | 79 | 31.6 | 188 | 37.2 |  |
| Unknown | 2 | 0.8 | 1 | 0.2 |  |
| Dukes’ Stage |  |  |  |  | 0.848 |
| A | 35 | 14 | 75 | 14.8 |  |
| B | 129 | 51.6 | 271 | 53.6 |  |
| C1 | 54 | 21.6 | 99 | 19.6 |  |
| C2 | 29 | 11.6 | 52 | 10.3 |  |
| Undetermined/unknown | 3 | 1.2 | 9 | 1.8 |  |
| Neoadjuvant Treatment |  |  |  |  | 0.749 |
| Yes | 46 | 18.4 | 99 | 19.6 |  |
| No | 200 | 80 | 404 | 79.8 |  |
| Unknown | 4 | 1.6 | 3 | 0.6 |  |
| Adjuvant Treatment1 |  |  |  |  | 0.196 |
| Yes | 79 | 31.6 | 185 | 36.6 |  |
| No | 169 | 67.6 | 320 | 63.2 |  |
| Unknown | 2 | 0.8 | 1 | 0.2 |  |
| Stoma |  |  |  |  | 0.536 |
| No | 155 | 62 | 324 | 64.0 |  |
| Yes | 92 | 36.8 | 174 | 34.4 |  |
| Unknown | 3 | 1.2 | 8 | 1.6 |  |
| Co-morbidities2 |  |  |  |  | 0.486 |
| None | 39 | 15.6 | 131 | 25.9 |  |
| One | 46 | 18.4 | 151 | 29.8 |  |
| Two | 43 | 17.2 | 103 | 20.4 |  |
| Three or more | 24 | 9.6 | 81 | 16.0 |  |
| Did not participate at 3m | 97 | 38.8 | 31 | 6.1 |  |
| Unknown | 1 | 0.4 | 9 | 1.8 |  |

*Among the 526 participants who completed the SCNS at 15 months, 20 patients did not complete measures at baseline and, therefore, were excluded from the analysis of baseline factors of SCNS completion at 15 months. Among 346 patients who did not complete the SCNS at 15 months, 96 patients were excluded due to their incompletion of the baseline questionnaire.

1 Chi-squared test; not adjusted for missing values.

2 excluding patients who did not participate at 3 months.

**Table 1.2**: Comparison of patient reported health and psychosocial characteristics at diagnosis (or 3 months post-surgery for EORTC QLQ-C30 and EORTC QLQ-CR29 subscale scores) according to whether the SCNS was completed at 15 months.

| **Continuous variables^1^** | ***Mean*** | ***SD*** | ***Skewedness*** | **Used cut-off for a binary categorisation** | **Non-completers (N=250)** | | **Completers (N=506)** | | **P-value^2^** |
| --- | --- | --- | --- | --- | --- | --- | --- | --- | --- |
|  |  |  |  |  | **N** | ***%*** | **N** | ***%*** |  |
| EQ-5D utility score | 0.78 | 0.22 | -1.42 | score is equal 1 | 63 | 25.2 | 165 | 32.6 | > 0.05 |
| QLACS GSS | 70.75 | 24.77 | 0.65 | *N/A (Mean and SD)* | *72.88* | *25.3* | *69.77* | *24.5* | *> 0.1* |
| PWI-A | 78.86 | 16.31 | -1.34 | score >= 70 | 173 | 69.2 | 397 | 78.5 | 0.023 |
| STAI | 36.50 | 12.67 | 0.66 | score >= 40 | 102 | 40.8 | 190 | 37.5 | > 0.1 |
| CES-D | 13.33 | 9.04 | 0.88 | score >= 20 | 65 | 26.0 | 93 | 18.4 | 0.009 |
| MOS-SSS | 80.88 | 20.95 | -1.46 | score = 100 | 52 | 20.8 | 108 | 21.3 | > 0.1 |
| Self-efficacy (LORIG) | 7.44 | 1.91 | -0.80 | *four-categorical version:* | | | | | 0.018 |
|  |  |  |  | score 1-4 | 38 | 15.2 | 46 | 9.1 |  |
|  |  |  |  | score 5-7 | 57 | 22.8 | 111 | 21.9 |  |
|  |  |  |  | score 7-9 | 87 | 34.8 | 230 | 45.5 |  |
|  |  |  |  | score 9=10 | 55 | 22.0 | 117 | 23.1 |  |
| PANAS negative | 8.64 | 3.68 | 1.27 | score <= 8 (median) | 137 | 54.8 | 304 | 60.1 | > 0.1 |
| PANAS positive | 18.07 | 4.50 | -0.63 | score <= 19 (median) | 145 | 58.0 | 280 | 55.3 | > 0.05 |
| C30 global health/QoL | 69.28 | 20.21 | -0.75 | *N/A (Mean and SD)* | *69.28* | *23.2* | *69.29* | *19.1* | *> 0.1* |
| C30 physical functioning | 75.50 | 21.87 | -0.89 | having a problem | 81 | 32.4 | 236 | 46.6 | > 0.1 |
| C30 role functioning | 69.21 | 31.19 | -0.76 | having a problem | 47 | 18.8 | 146 | 28.9 | > 0.1 |
| C30 emotional functioning | 77.76 | 21.29 | -1.07 | having a problem | 37 | 14.8 | 108 | 21.3 | > 0.1 |
| C30 cognitive functioning | 75.68 | 23.19 | -0.97 | having a problem | 29 | 11.6 | 87 | 17.2 | > 0.1 |
| C30 social functioning | 70.04 | 29.54 | -0.80 | having a problem | 47 | 18.8 | 145 | 28.7 | > 0.1 |
| C30 fatigue | 37.31 | 24.93 | 0.62 | having a problem | 59 | 23.6 | 190 | 37.5 | > 0.1 |
| C30 nausea & vomiting | 8.27 | 15.80 | 2.43 | having a problem | 9 | 3.6 | 47 | 9.3 | > 0.1 |
| C30 pain | 26.55 | 24.58 | 0.86 | having a problem | 25 | 10.0 | 77 | 15.2 | > 0.1 |
| C30 dyspnoea | 16.91 | 24.70 | 1.48 | having a problem | 18 | 7.2 | 40 | 7.9 | > 0.1 |
| C30 insomnia | 32.85 | 31.30 | 0.66 | having a problem | 34 | 13.6 | 126 | 24.9 | > 0.1 |
| C30 appetite loss | 15.53 | 26.01 | 1.76 | having a problem | 12 | 4.8 | 49 | 9.7 | > 0.1 |
| C30 constipation | 13.94 | 24.25 | 1.78 | having a problem | 17 | 6.8 | 41 | 8.1 | > 0.1 |
| C30 diarrhoea | 18.02 | 26.82 | 1.43 | having a problem | 19 | 7.6 | 60 | 11.9 | > 0.1 |
| C30 financial difficulties | 14.92 | 27.22 | 1.79 | having a problem | 18 | 7.2 | 60 | 11.9 | > 0.1 |
| CR29 body image | 78.36 | 26.69 | -1.42 | having a problem | 35 | 14.0 | 112 | 22.1 | > 0.1 |
| CR29 anxiety | 62.03 | 27.89 | -0.52 | having a problem | 40 | 16.0 | 128 | 25.3 | > 0.1 |
| CR29 weight | 75.20 | 28.35 | -1.01 | having a problem | 22 | 8.8 | 80 | 15.8 | > 0.1 |
| CR29 sexual interest | 26.64 | 28.76 | 0.80 | having no interest | 102 | 40.8 | 345 | 68.2 | > 0.1 |
| CR29 urinary frequency | 36.53 | 24.74 | 0.24 | having a problem | 75 | 30.0 | 211 | 41.7 | > 0.1 |
| CR29 blood & mucus in stool | 4.07 | 12.14 | 4.73 | having a problem | 3 | 1.2 | 15 | 3.0 | > 0.1 |
| CR29 urinary incontinence | 7.72 | 17.31 | 2.61 | having a problem | 4 | 1.6 | 12 | 2.4 | > 0.1 |
| CR29 dysuria | 4.11 | 13.20 | 3.85 | having a problem | 1 | 0.4 | 8 | 1.6 | > 0.1 |
| CR29 abdominal pain | 13.52 | 21.20 | 1.61 | having a problem | 10 | 4.0 | 24 | 4.7 | > 0.1 |
| CR29 buttock pain | 14.15 | 24.72 | 1.81 | having a problem | 10 | 4.0 | 48 | 9.5 | > 0.1 |
| CR29 bloating | 17.29 | 23.84 | 1.38 | having a problem | 9 | 3.6 | 43 | 8.5 | > 0.1 |
| CR29 dry mouth | 23.72 | 28.32 | 1.05 | having a problem | 27 | 10.8 | 74 | 14.6 | > 0.1 |
| CR29 hair loss | 5.76 | 15.22 | 2.89 | having a problem | 4 | 1.6 | 12 | 2.4 | > 0.1 |
| CR29 taste | 19.65 | 29.39 | 1.37 | having a problem | 20 | 8.0 | 78 | 15.4 | > 0.1 |
| CR29 stoma care problems | 17.67 | 25.65 | 1.49 | *three-categorical version:* | | | | | > 0.1 |
|  |  |  |  | having no stoma | 158 | 63.2 | 341 | 67.4 |  |
|  |  |  |  | stoma, no problem | 42 | 16.8 | 138 | 27.3 |  |
|  |  |  |  | stoma and problem | 7 | 2.8 | 13 | 2.6 |  |

^1^ Due to a skewed distribution in most of the psychosocial measures and EORTC subscales, all scores, with the exception of QLACS-GSS and the QLQ-C30 global health/QoL score, were converted into categorical covariates using the following cut-offs: 1 for EQ-5D full health, 70 for PWI-A, 20 for CES-D, 40 for STAI, and four groups for Lorig. Additionally, a median score was used to dichotomise both scores of PANAS and the maximum score of 100 was applied as a cut-off for MOS SSS due to its highly-skewed distribution. As for QLQ-C30 and QLQ-CR29, if any of the items on each subscale indicated a moderate or severe problem, the subscale was identified as being clinically significant and scored one; otherwise it was scored as zero. The QLQ-CR29 subscale of stoma care problem was combined with the stoma status into three categories. QLQ-CR29 sexual interest was combined into one measure for both sexes with missing values categorised as a third category. However, due to high levels of missing values (>10%) in other QLQ-CR29 subscales, the following QLQ-CR29 covariates were excluded from the analyses: stool frequency, flatulence, faecal incontinence, sore skin, and embarrassment. QLQ-CR29 impotence (men) and dyspareunia (women) were also excluded in subsequent analyses due to inability to be accounted for in the same regression model. The QLQ-CR29 sexual interest subscale score was combined for men and women

^2^ Chi-squared test was used to calculate p-values for all categorical variables; T-test was used for QLACS-GSS and C30 global health/QoL; statistics for EORTC C30 and CR29 exclude those who did not participate at 3 months (N=128)

*Abbreviations:* SCNS= Supportive Care Needs Survey; EQ-5D VAS = The EuroQol 5 dimension Visual Analogue Scale; QLACS GSS= Quality of Life in Adult Cancer Survivors Generic Summary Score; PWI-A = Psychological Wellbeing Index Adult Form; STAI = State Trait Anxiety Inventory; CES-D = Centre for Epidemiologic Studies Depression Scale; MOS- SSS = The MOS Social Support Survey; PANAS = Positive and Negative Affect Scale; QLQ-C30 = EORTC QLQ-Core 30, QLQ-CR29 = EORTC QLQ-CR29;

**Table 1.3**: Multivariable logistic regression model with significant estimates of patient’s likelihood to complete the SCNS at 15 months, baseline covariates

| **Baseline covariates** | **OR** | **SE** | **CI 95%** | |
| --- | --- | --- | --- | --- |
| **Age** |  |  |  |  |
| <50 | 1.00 |  |  |  |
| 51-60 | 1.37 | 0.52 | 0.65 | 2.87 |
| 61-70 | 1.45 | 0.50 | 0.74 | 2.84 |
| 71-80 | 0.98 | 0.34 | 0.50 | 1.94 |
| >80 | 0.42* | 0.17 | 0.19 | 0.92 |
| **Domestic status** |  |  |  |  |
| Married | 1.00 |  |  |  |
| Living with a partner | 0.50* | 0.17 | 0.25 | 0.98 |
| Widowed | 0.61* | 0.14 | 0.38 | 0.96 |
| Divorced / Separated | 1.07 | 0.33 | 0.58 | 1.96 |
| Single | 0.51* | 0.16 | 0.27 | 0.96 |

* *p*-value [0.03-0.04]
