# Supplementary material for: Supportive care needs of patients following treatment for colorectal cancer: risk factors for unmet needs and the association between unmet needs and health-related quality of life—results from the ColoREctal Wellbeing (CREW) study
Source: J Cancer Surviv. 2019 Sep 11;13(6):899–909. doi: 10.1007/s11764-019-00805-6 (PMC6881415; doi:10.1007/s11764-019-00805-6)
Supplement: Supplementary file 2 — (DOCX 23 kb) [file 11764_2019_805_MOESM2_ESM.docx]

**Supplementary Material 2**: Prevalence of unmet need for individual SCNS items at 15 months and 24 months post-surgery

|  |  | **15 months**  N=526 | **24 months**  N=510 |
| --- | --- | --- | --- |
| Item | Domain | N (%) | N (%) |
| **1. Pain** | **Physical and Daily Living** |  |  |
| Not applicable |  | 392 (74.5%) | 381 (74.7%) |
| Satisfied |  | 59 (11.2%) | 35 (6.8%) |
| Low need |  | 31 (5.9%) | 37 (7.3%) |
| Moderate need |  | 19 (3.6%) | 32 (6.3%) |
| High need |  | 10 (1.9%) | 4 (0.8%) |
| *Missing* |  | *15 (2.9%)* | *21 (4.1%)* |
| **2. Lack of energy/tiredness** | **Physical and Daily Living** |  |  |
| Not applicable |  | 305 (58.0%) | 315 (61.8%) |
| Satisfied |  | 88 (16.7%) | 61 (12.0%) |
| Low need |  | 71 (13.5%) | 60 (11.8%) |
| Moderate need |  | 34 (6.5%) | 40 (7.8%) |
| High need |  | 14 (2.7%) | 12 (2.4%) |
| *Missing* |  | *14 (2.7%)* | *22 (4.3%)* |
| **3. Feeling unwell** | **Physical and Daily Living** |  |  |
| Not applicable |  | 399 (75.9%) | 393 (77.1%) |
| Satisfied |  | 44 (8.4%) | 35 (6.9%) |
| Low need |  | 36 (6.8%) | 38 (7.5%) |
| Moderate need |  | 19 (3.6%) | 18 (3.5%) |
| High need |  | 4 (0.8%) | 1 (0.2%) |
| *Missing* |  | *24 (4.6%)* | *25 (4.9%)* |
| **4. Work around the home** | **Physical and Daily Living** |  |  |
| Not applicable |  | 365 (69.4%) | 348 (68.2%) |
| Satisfied |  | 74 (14.1%) | 68 (13.3%) |
| Low need |  | 35 (6.7%) | 44 (8.6%) |
| Moderate need |  | 25 (4.8%) | 15 (2.9%) |
| High need |  | 7 (1.3%) | 12 (2.4%) |
| *Missing* |  | *20 (3.8%)* | *23 (4.5%)* |
| **5. Not being able to do the things you used to do** | **Physical and Daily Living** |  |  |
| Not applicable |  | 322 (61.2%) | 324 (63.5%) |
| Satisfied |  | 78 (14.8%) | 56 (11.0%) |
| Low need |  | 59 (11.2%) | 59 (11.6%) |
| Moderate need |  | 42 (8.0%) | 32 (6.3%) |
| High need |  | 17 (3.2%) | 22 (4.3%) |
| *Missing* |  | *8 (1.5%)* | *17 (3.3%)* |
| **6. Anxiety** | **Psychological** |  |  |
| Not applicable |  | 368 (70.0%) | 345 (67.7%) |
| Satisfied |  | 58 (11.0%) | 52 (10.2%) |
| Low need |  | 50 (9.5%) | 52 (10.2%) |
| Moderate need |  | 22 (4.2%) | 25 (4.9%) |
| High need |  | 9 (1.7%) | 16 (3.1%) |
| *Missing* |  | *19 (3.6%)* | *20 (3.9%)* |
| **7. Feeling down or depressed** | **Psychological** |  |  |
| Not applicable |  | 352 (66.9%) | 345 (67.7%) |
| Satisfied |  | 68 (12.9%) | 54 (10.6%) |
| Low need |  | 65 (12.4%) | 46 (9.0%) |
| Moderate need |  | 17 (3.2%) | 37 (7.3%) |
| High need |  | 7 (1.3%) | 13 (2.6%) |
| *Missing* |  | *17 (3.2%)* | *15 (2.9%)* |
| **8. Feelings of sadness** | **Psychological** |  |  |
| Not applicable |  | 364 (69.2%) | 352 (69.0%) |
| Satisfied |  | 63 (12.0%) | 53 (10.4%) |
| Low need |  | 58 (11.0%) | 38 (7.5%) |
| Moderate need |  | 17 (3.2%) | 37 (7.3%) |
| High need |  | 6 (1.1%) | 11 (2.2%) |
| *Missing* |  | *18 (3.4%)* | *19 (3.7%)* |
| **9. Fears about cancer spreading** | **Psychological** |  |  |
| Not applicable |  | 280 (53.2%) | 284 (55.7%) |
| Satisfied |  | 108 (20.5%) | 68 (13.3%) |
| Low need |  | 72 (13.7%) | 83 (16.3%) |
| Moderate need |  | 32 (6.1%) | 33 (6.5%) |
| High need |  | 18 (3.4%) | 23 (4.5%) |
| *Missing* |  | *16 (3.0%)* | *19 (3.7%)* |
| **10. Worry that the results of treatment are beyond your control** | **Psychological** |  |  |
| Not applicable |  | 359 (70.4%) | 365 (71.6%) |
| Satisfied |  | 71 (13.9%) | 57 (11.2%) |
| Low need |  | 46 (9.0%) | 33 (6.5%) |
| Moderate need |  | 23 (4.5%) | 27 (5.3%) |
| High need |  | 11 (2.2%) | 13 (2.6%) |
| *Missing* |  | *16 (3.0%)* | *15 (2.9%)* |
| **11. Uncertainty** | **Psychological** |  |  |
| Not applicable |  | 319 (60.6%) | 322 (63.1%) |
| Satisfied |  | 79 (15.0%) | 65 (12.8%) |
| Low need |  | 68 (12.9%) | 64 (12.6%) |
| Moderate need |  | 34 (6.5%) | 26 (5.1%) |
| High need |  | 12 (2.3%) | 17 (3.3%) |
| *Missing* |  | *14 (2.7%)* | *16 (3.1%)* |
| **12. Learning to feel in control** | **Psychological** |  |  |
| Not applicable |  | 333 (63.3%) | 330 (64.7%) |
| Satisfied |  | 104 (19.8%) | 98 (19.2%) |
| Low need |  | 51 (9.7%) | 36 (7.1%) |
| Moderate need |  | 17 (3.2%) | 27 (5.3%) |
| High need |  | 3 (0.6%) | 5 (1.0%) |
| *Missing* |  | *18 (3.4%)* | *14 (2.8)* |
| **13. Keeping a positive outlook** | **Psychological** |  |  |
| Not applicable |  | 342 (65.0%) | 322 (63.1%) |
| Satisfied |  | 117 (22.2%) | 115 (22.6%) |
| Low need |  | 35 (6.7%) | 32 (6.3%) |
| Moderate need |  | 14 (2.7%) | 18 (3.5%) |
| High need |  | 3 (0.6%) | 9 (1.8%) |
| *Missing* |  | *15 (2.9%)* | *14 (2.8%)* |
| **14. Feelings about death and dying** | **Psychological** |  |  |
| Not applicable |  | 360 (68.4%) | 356 (69.8%) |
| Satisfied |  | 76 (14.4%) | 64 (12.6%) |
| Low need |  | 49 (9.3%) | 47 (9.2%) |
| Moderate need |  | 20 (3.8%) | 18 (3.5%) |
| High need |  | 6 (1.1%) | 10 (2.0%) |
| *Missing* |  | *15 (2.9%)* | *15 (2.9%)* |
| **15. Changes in sexual feelings** | **Sexuality** |  |  |
| Not applicable |  | 376 (71.5%) | 371 (72.8%) |
| Satisfied |  | 56 (10.6%) | 49 (9.6%) |
| Low need |  | 33 (6.3%) | 31 (6.1%) |
| Moderate need |  | 21 (4.0%) | 14 (2.8%) |
| High need |  | 18 (3.4%) | 20 (3.9%) |
| *Missing* |  | *22 (4.2%)* | *25 (4.9%)* |
| **16. Changes in sexual relationships** | **Sexuality** |  |  |
| Not applicable |  | 392 (74.5%) | 384 (75.3%) |
| Satisfied |  | 46 (8.7%) | 41 (8.0%) |
| Low need |  | 32 (6.1%) | 22 (4.3%) |
| Moderate need |  | 18 (3.4%) | 17 (3.3%) |
| High need |  | 16 (3.0%) | 18 (3.5%) |
| *Missing* |  | *21 (4.0%)* | *28 (5.5)* |
| **17. Concerns about the worries of those close to you** | **Psychological** |  |  |
| Not applicable |  | 312 (59.3%) | 307 (60.2%) |
| Satisfied |  | 92 (17.5%) | 87 (17.1%) |
| Low need |  | 64 (12.2%) | 52 (10.2%) |
| Moderate need |  | 32 (6.1%) | 27 (5.3%) |
| High need |  | 12 (2.3%) | 22 (4.3%) |
| *Missing* |  | *14 (2.7%)* | *15 (2.9%)* |
| **18. More choice about which cancer specialist you see** | **Patient care and support** |  |  |
| Not applicable |  | 408 (77.6%) | 395 (77.5%) |
| Satisfied |  | 82 (15.6%) | 64 (12.5%) |
| Low need |  | 13 (2.5%) | 13 (2.6%) |
| Moderate need |  | 5 (1.0%) | 16 (3.1%) |
| High need |  | 5 (1.0%) | 7 (1.4%) |
| *Missing* |  | *13 (2.5%)* | *15 (2.9%)* |
| **19. More choice about which hospital you attend** | **Patient care and support** |  |  |
| Not applicable |  | 411 (78.1%) | 395 (77.5%) |
| Satisfied |  | 75 (14.3%) | 73 (14.3%) |
| Low need |  | 10 (1.9%) | 14 (2.8%) |
| Moderate need |  | 8 (1.5%) | 7 (1.4%) |
| High need |  | 9 (1.7%) | 6 (1.2%) |
| *Missing* |  | *13 (2.5%)* | *15 (2.9%)* |
| **20. Reassurance by medical staff** | **Patient care and support** |  |  |
| Not applicable |  | 362 (68.8%) | 352 (69.0%) |
| Satisfied |  | 120 (22.8%) | 93 (18.2%) |
| Low need |  | 21 (4.0%) | 26 (5.1%) |
| Moderate need |  | 12 (2.3%) | 16 (3.1%) |
| High need |  | 3 (0.6%) | 6 (1.2%) |
| *Missing* |  | *8 (1.5%)* | *17 (3.3%)* |
| **21. Hospital staff promptly attending physical needs** | **Patient care and support** |  |  |
| Not applicable |  | 403 (76.6%) | 391 (76.7%) |
| Satisfied |  | 96 (18.3%) | 73 (14.3%) |
| Low need |  | 9 (1.7%) | 15 (2.9%) |
| Moderate need |  | 5 (1.0%) | 7 (1.4%) |
| High need |  | 4 (0.8%) | 3 (0.6%) |
| *Missing* |  | *9 (1.7%)* | *21 (4.1%)* |
| **22. Hospital staff acknowledging feelings and emotional needs** | **Patient care and support** |  |  |
| Not applicable |  | 383 (72.8%) | 375 (73.5%) |
| Satisfied |  | 117 (22.2%) | 83 (16.3%) |
| Low need |  | 6 (1.1%) | 19 (3.7%) |
| Moderate need |  | 10 (1.9%) | 7 (1.4%) |
| High need |  | 2 (0.4%) | 6 (1.2%) |
| *Missing* |  | *8 (1.5%)* | 20 (3.9%) |
| **23. Written information about important aspects of care** | **Health system and information needs** |  |  |
| Not applicable |  | 396 (75.3%) | 377 (73.9%) |
| Satisfied |  | 97 (18.4%) | 74 (14.5%) |
| Low need |  | 13 (2.5%) | 15 (2.9%) |
| Moderate need |  | 7 (1.3%) | 12 (2.4%) |
| High need |  | 2 (0.4%) | 9 (1.8%) |
| *Missing* |  | *11 (2.1%)* | *23 (4.5%)* |
| **24. Written information about managing illness and side-effects at home** | **Health system and information needs** |  |  |
| Not applicable |  | 418 (81.2%) | 401 (78.6%) |
| Satisfied |  | 74 (14.4%) | 56 (11.0%) |
| Low need |  | 13 (2.5%) | 17 (3.3%) |
| Moderate need |  | 7 (1.4%) | 8 (1.6%) |
| High need |  | 3 (0.6%) | 8 (1.6%) |
| *Missing* |  | *11 (2.1%)* | *13 (3.9%)* |
| **25. Explanations of tests** | **Health system and information needs** |  |  |
| Not applicable |  | 366 (69.6%) | 344 (67.5%) |
| Satisfied |  | 122 (23.2%) | 103 (20.2%) |
| Low need |  | 13 (2.5%) | 18 (3.5%) |
| Moderate need |  | 10 (1.9%) | 14 (2.8%) |
| High need |  | 6 (1.1%) | 9 (1.8%) |
| *Missing* |  | *9 (1.7%)* | *22 (4.3)* |
| **26. Information about benefits and side-effects of treatment** | **Health system and information needs** |  |  |
| Not applicable |  | 383 (72.8%) | 380 (74.5%) |
| Satisfied |  | 108 (20.5%) | 83 (16.3%) |
| Low need |  | 12 (2.3%) | 14 (2.8%) |
| Moderate need |  | 8 (1.5%) | 3 (0.6%) |
| High need |  | 6 (1.1%) | 9 (1.8%) |
| *Missing* |  | *9 (1.7%)* | *21 (4.1)* |
| **27. Information about test results** | **Health system and information needs** |  |  |
| Not applicable |  | 300 (57.0%) | 292 (57.3%) |
| Satisfied |  | 174 (33.1%) | 147 (28.8%) |
| Low need |  | 16 (3.0%) | 18 (3.5%) |
| Moderate need |  | 13 (2.5%) | 12 (2.4%) |
| High need |  | 14 (2.7%) | 20 (3.9%) |
| *Missing* |  | *9 (1.7%)* | *21 (4.1%)* |
| **28. Information about cancer under control / diminishing** | **Health system and information needs** |  |  |
| Not applicable |  | 338 (64.3%) | 318 (62.3%) |
| Satisfied |  | 144 (27.4%) | 127 (24.9%) |
| Low need |  | 16 (3.0%) | 20 (3.9%) |
| Moderate need |  | 7 (1.3%) | 12 (2.4%) |
| High need |  | 10 (1.9%) | 14 (2.8%) |
| *Missing* |  | *11 (2.1%)* | *19 (3.7%)* |
| **29. Informed about things to help yourself** | **Health system and information needs** |  |  |
| Not applicable |  | 356 (67.7%) | 361 (70.8%) |
| Satisfied |  | 121 (23.0%) | 82 (16.0%) |
| Low need |  | 25 (4.8%) | 13 (2.6%) |
| Moderate need |  | 9 (1.7%) | 14 (2.8%) |
| High need |  | 5 (1.0%) | 12 (2.4%) |
| *Missing* |  | *10 (1.9%)* | *28 (5.5%)* |
| **30. Access to professional counselling** | **Health system and information needs** |  |  |
| Not applicable |  | 392 (74.5%) | 386 (75.7%) |
| Satisfied |  | 95 (18.1%) | 63 (12.4%) |
| Low need |  | 11 (2.1%) | 18 (3.5%) |
| Moderate need |  | 14 (2.7%) | 13 (2.6%) |
| High need |  | 4 (0.8%) | 9 (1.8%) |
| *Missing* |  | *10 (1.9%)* | *21 (4.1%)* |
| **31. Information about sexual relationships** | **Sexuality** |  |  |
| Not applicable |  | 428 (81.4%) | 412 (80.8%) |
| Satisfied |  | 43 (8.2%) | 26 (5.1%) |
| Low need |  | 17 (3.2%) | 20 (3.9%) |
| Moderate need |  | 17 (3.2%) | 14 (2.8%) |
| High need |  | 7 (1.3%) | 12 (2.4%) |
| *Missing* |  | *14 (2.7%)* | *26 (5.1%)* |
| **32. Treated like a person** | **Health system and information needs** |  |  |
| Not applicable |  | 357 (67.9%) | 346 (67.8%) |
| Satisfied |  | 138 (26.2%) | 111 (21.8%) |
| Low need |  | 8 (1.5%) | 17 (3.3%) |
| Moderate need |  | 5 (1.0%) | 8 (1.6%) |
| High need |  | 6 (1.1%) | 9 (1.8%) |
| *Missing* |  | *12 (2.3%)* | *19 (3.7%)* |
| **33. Treated in a hospital / clinic that is physically pleasant** | **Health system and information needs** |  |  |
| Not applicable |  | 357 (69.2%) | 339 (66.5%) |
| Satisfied |  | 139 (26.9%) | 124 (24.3%) |
| Low need |  | 9 (1.7%) | 15 (2.9%) |
| Moderate need |  | 2 (0.4%) | 4 (0.8%) |
| High need |  | 9 (1.7%) | 8 (1.6%) |
| *Missing* |  | *10 (1.9%)* | *20 (3.9%)* |
| **34. Having a member of staff to talk to** | **Health system and information needs** |  |  |
| Not applicable |  | 337 (67.9) | 321 (62.9%) |
| Satisfied |  | 144 (27.4%) | 127 (24.9%) |
| Low need |  | 15 (2.9%) | 22 (4.3%) |
| Moderate need |  | 8 (1.5%) | 7 (1.4%) |
| High need |  | 13 (2.5%) | 16 (3.1%) |
| *Missing* |  | *9 (1.7%)* | *17 (3.3%)* |
