# Supplementary material for: Supportive care needs of patients following treatment for colorectal cancer: risk factors for unmet needs and the association between unmet needs and health-related quality of life—results from the ColoREctal Wellbeing (CREW) study
Source: J Cancer Surviv. 2019 Sep 11;13(6):899–909. doi: 10.1007/s11764-019-00805-6 (PMC6881415; doi:10.1007/s11764-019-00805-6)
Supplement: Supplementary file 3 — (DOCX 17 kb) [file 11764_2019_805_MOESM3_ESM.docx]

**Supplementary Material 3**: Within-blocks logistic regression models of SCNS domains

| **Baseline covariates** | **SCNS domains at 15 months as outcomes** | | | | |
| --- | --- | --- | --- | --- | --- |
|  | **Physical and Daily Living** | **Psychological** | **Sexuality** | **Patient Care and Support** | **Health system and information needs** |
| **N with no missing in outcome** | **473** | **471** | **466** | **473** | **470** |
| *Block 1: Socio-demographic covariates* | *Model 1.1* | *Model 2.1* | *Model 3.1* | *Model 4.1* | *Model 5.1* |
| Female (ref: male) |  | *None are statistically significant (incl. univariate analysis)* | 0.23** |  | *None are statistically significant (incl. univariate analysis)* |
| Aged 61-70 (ref: aged <=60) | 0.39* |  | 0.27** | 0.54 |  |
| Aged 71+ (ref: aged <=60) | 0.40* |  | 0.37* | 0.31* |  |
| Employed (ref: unemployed/retired) | 3.65** |  |  |  |  |
| No partner (ref: married/cohabiting) |  |  |  |  |  |
| *Block 2: Clinical covariates* | *Model 1.2* | *Model 2.2* | *Model 3.2* | *Model 4.2* | *Model 5.2* |
| 1+ comorbidity (ref: none) | 4.48*** |  |  |  |  |
| Rectum T-site group (ref: Colon) |  |  |  |  |  |
| Dukes stage C1-C2 (ref: stage A-B) |  |  |  |  |  |
| Neo-adjuvant CT/RT/both (ref: none) |  | 2.49** |  | 3.81*** | 2.18* |
| Adjuvant CT/RT/both (ref: none) |  | 3.19*** |  |  | 2.34** |
| Stoma (ref: no stoma) | 2.88*** |  | 0.24*** |  |  |
| *Block 3. CREW-specific scales* | *Model 1.3* | *Model 2.3* | *Model 3.3* | *Model 4.3* | *Model 5.3* |
| PWI-A (ref: below 70) |  |  |  |  |  |
| STAI (ref: below 40) |  |  |  |  |  |
| CES-D (ref: below 20) |  |  |  |  |  |
| EQ-5D full health (ref: ‘not’, score <1) | 0.39** | 0.45* |  |  |  |
| QLACS GSS *(a score*) |  | 1.03*** |  | 1.02** | 1.02*** |
| MOS full support of 100 (ref: not) |  |  |  |  |  |
| Good/High Self-efficacy (ref: low/moderate confidence) | 2.29** |  | 3.76*** |  |  |
| PANAS negative (ref: below median) |  |  |  |  |  |
| PANAS positive (ref: below median) |  |  |  |  |  |
| *Block 4. QLQ-C30 subscales (ref: no clinically significant problem)* | *Model 1.4* | *Model 2.4* | *Model 3.4* | *Model 4.4* | *Model 5.4* |
| Health-related QoL *(a score*) | 0.97** |  | 0.97** | 0.97** |  |
| physical functioning |  |  |  |  |  |
| role functioning | 2.72** | 2.41** |  |  |  |
| emotional functioning |  | 2.48** |  |  |  |
| cognitive functioning |  |  |  |  |  |
| social functioning |  | 2.50** |  | 2.78* | 3.29*** |
| fatigue | 2.74** |  |  |  |  |
| nausea & vomiting |  |  |  |  |  |
| pain |  |  |  |  |  |
| dyspnoea |  |  |  |  |  |
| insomnia |  |  |  |  |  |
| appetite loss |  |  |  |  |  |
| constipation |  |  |  |  |  |
| diarrhoea |  |  |  |  |  |
| financial difficulties |  |  | 2.53* |  |  |
| *Block 5. QLQ-CR29 subscales (ref: no clinically significant problem)* | *Model 1.5* | *Model 2.5* | *Model 3.5* | *Model 4.5* | *Model 5.5* |
| body image | 2.64** | 2.58** | 4.69*** | 5.65*** | 4.90*** |
| anxiety | 2.21* | 2.03* |  | 3.20** |  |
| weight |  |  |  |  |  |
| sexual interest |  |  |  |  |  |
| urinary frequency | 1.80* |  |  |  |  |
| stool frequency |  |  |  |  |  |
| buttock pain | 4.73*** | 2.75** | 5.47*** |  |  |
| bloating |  |  |  |  |  |
| dry mouth |  |  |  |  |  |
| taste |  | 2.37** |  |  |  |
|  |  |  |  |  |  |
| *Note*: those covariates which have empty cells for the estimates were statistically insignificant (p>0.05).  * p<.05; ** p<.01; *** p<.001 | | | | | |
