# Supplementary material for: Supportive care needs of patients following treatment for colorectal cancer: risk factors for unmet needs and the association between unmet needs and health-related quality of life—results from the ColoREctal Wellbeing (CREW) study
Source: J Cancer Surviv. 2019 Sep 11;13(6):899–909. doi: 10.1007/s11764-019-00805-6 (PMC6881415; doi:10.1007/s11764-019-00805-6)
Supplement: Supplementary file 4 — (DOCX 14 kb) [file 11764_2019_805_MOESM4_ESM.docx]

**Supplementary Material 4**: Multivariable linear regression models of QLQ-C30 Global health/QoL at 15 months, significant covariates at 15 months per each thematic block

| **Independent (at 15 months) variables** | **Coefficient** | **( OR 95% CI)** |
| --- | --- | --- |
| ***Model 1: Socio-demographic block*** |  |  |
| Age group (ref: aged 60 or younger) | 0.00 |  |
| Aged 61-70 | 6.97** | (2.44; 11.50) |
| Aged 71 or older | 4.74 | (-0.19; 9.68) |
| Employment status (ref: employed) | 0.00 |  |
| Unemployed / retired | -4.88* | (-9.25; -0.51) |
| Domestic status (ref: married / cohabiting) | 0.00 |  |
| Single / never married / divorced / widowed | -4.54** | (-7.95; -1.13) |
| ***Model 2: Clinical block*** |  |  |
| Comorbidities (ref: none) | 0.00 |  |
| Yes, at least one | -10.33*** | (-13.64 ; -7.01) |
| Neo-adjuvant therapy (ref: none) |  |  |
| Yes, any (chemotherapy / radiotherapy /both) | -5.80** | (-10.01; -1.59) |
| Stoma (ref: no) |  |  |
| Yes, had a stoma | -4.29* | (-7.83; -0.74) |
| Recurrence (ref: no) |  |  |
| Yes, had a recurrence by 15 months | -5.91* | (-11.06 ; -0.77) |
| ***Model 3: Negative life events block*** |  |  |
| Had any negative life event in the last 6 months (ref: none) |  |  |
| Yes, at least one | -7.37*** | (-10.52; -4.23) |
| ***Model 4: SCNS needs block*** |  |  |
| Physical and daily living needs (ref: no need / low level) |  |  |
| Yes, high level of this domain of needs | -16.93*** | (-21.29; -12.57) |
| Psychological needs (ref: no need / low level) |  |  |
| Yes, high level of this domain of needs | -8.29*** | (-12.56 ; -4.02) |
| Health system and information needs (ref: no need / low level) |  |  |
| Yes, high level of this domain of needs | -6.99** | (-11.56; -2.41) |

* p<0.05; ** p<0.01; *** p<0.001
