# Supplementary material for: Supportive care needs of patients following treatment for colorectal cancer: risk factors for unmet needs and the association between unmet needs and health-related quality of life—results from the ColoREctal Wellbeing (CREW) study
Source: J Cancer Surviv. 2019 Sep 11;13(6):899–909. doi: 10.1007/s11764-019-00805-6 (PMC6881415; doi:10.1007/s11764-019-00805-6)
Supplement: Supplementary file 5 — (DOCX 13 kb) [file 11764_2019_805_MOESM5_ESM.docx]

**Supplementary Material 5**: Multivariable linear regression models of QLQ-C30 Global health/QoL at 15 months with separate models for each SCNS domain, significant covariates at 15 months

| **Independent Variables at 15 months** | SCNS domains (as covariates) | | | | |  | |
| --- | --- | --- | --- | --- | --- | --- | --- |
|  | Model 1: Physical and Daily Living | Model 2: Psycho-logical | Model 3: Sexuality | Model 4: Patient Care and Support | Model 5: Health System and Information |  |  |
| High level of the SCNS Domain (ref: low level) | -18.90*** | -14.74*** | -11.87*** | -14.34*** | -11.87*** |  |  |
| *Block 1: Socio-demographic covariates* |  |  |  |  |  |  |  |
| No partner (ref: married/cohabiting) | -4.74** | -4.91** | -5.48** | -5.07** | -4.79** |  |  |
| *Block 2: Clinical covariates* |  |  |  |  |  |  |  |
| 1+ comorbidity (ref: none) | -7.62*** | -9.16*** | -10.05*** | -10.55*** | -9.99*** |  |  |
| Neo-adjuvant CT/RT/both (ref: none) | -6.83*** | -5.22* | -5.87** | -5.52* | -5.98** |  |  |
| No stoma (ref: stoma) | *ns* | 3.68* | 4.10* | 3.94* | 3.81* |  |  |
| *Block 3: Life events* |  |  |  |  |  |  |  |
| At least one stressful life event (ref: none) | -5.41*** | -4.72** | -5.62*** | -6.16*** | -5.77*** |  |  |

Note: *ns* not significant; * p<0.05; ** p<0.01; *** p<0.001
