# Supplementary material for: Supportive care needs of patients following treatment for colorectal cancer: risk factors for unmet needs and the association between unmet needs and health-related quality of life—results from the ColoREctal Wellbeing (CREW) study
Source: J Cancer Surviv. 2019 Sep 11;13(6):899–909. doi: 10.1007/s11764-019-00805-6 (PMC6881415; doi:10.1007/s11764-019-00805-6)
Supplement: Supplementary file 6 — (DOCX 15 kb) [file 11764_2019_805_MOESM6_ESM.docx]

**Supplementary Material 6**: Multivariable linear regression models of QLQ-C30 Global health/QoL excluding seven ‘outliers’ who had a score less than 33

**Table 6.1**: Within-blocks linear regression models (Model 1-4)

| **Covariates at 15 months** | **Coef.** | **SE** |
| --- | --- | --- |
| *Model 1: Socio-demographic covariates* ^1^ |  |  |
| Domestic status (ref: married / cohabiting) | 0 |  |
| Single / never married / divorced / widowed | -3.67* | 1.61 |
| *Model 2: Clinical covariates* |  |  |
| Comorbidities (ref: none) | 0 |  |
| Yes, at least one | -9.44*** | 1.58 |
| Neo-adjuvant therapy (ref: none) | 0 |  |
| Yes, any (chemotherapy / radiotherapy /both) | -5.16* | 2.02 |
| Stoma (ref: no stoma) | 0 |  |
| Yes, stoma | 4.07* | 1.68 |
| *Model 3: Negative life events* |  |  |
| Had any negative life event in the last 6 months (ref: none) | 0 |  |
| Yes, at least one | -5.75*** | 1.50 |
| *Model 4: SCNS domains (ref in each: low needs)* |  |  |
| Physical and daily living needs (ref: no need / low level) | 0 |  |
| Yes, high level of this domain of needs | -14.88*** | 2.11 |
| Psychological needs (ref: no need / low level) | 0 |  |
| Yes, high level of this domain of needs | -6.86** | 2.06 |
| Health system and information needs (ref: no need / low level) | 0 |  |
| Yes, high level of this domain of needs | -6.39** | 2.23 |

^1^ Model 1 is adjusted for age of respondents;

* p<0.05; ** p<0.01; *** p<0.001

**Table 6.2**: Final linear regression model adjusted for all significant covariates from each thematic block above (Model 5)

| **Covariates at 15 months** | **Coef.** | **SE** |
| --- | --- | --- |
| Domestic status (ref: married / cohabiting) | 0 |  |
| Single / never married / divorced / widowed | -3.51* | 1.45 |
| Comorbidities (ref: none) | 0 |  |
| Yes, at least one | -6.76*** | 1.51 |
| Neo-adjuvant therapy (ref: none) | 0 |  |
| Yes, any (chemotherapy / radiotherapy /both) | -4.21* | 1.67 |
| Had any negative life event in the last 6 months (ref: none) | 0 |  |
| Yes, at least one | -3.59* | 1.41 |
| Physical and daily living needs (ref: no need / low level) | 0 |  |
| Yes, high level of this domain of needs | -13.43*** | 2.11 |
| Psychological needs (ref: no need / low level) | 0 |  |
| Yes, high level of this domain of needs | -6.23** | 2.07 |
| Health system and information needs (ref: no need / low level) | 0 |  |
| Yes, high level of this domain of needs | -5.76** | 2.21 |

*Note:* the model is adjusted for age of respondents; * p<0.05; ** p<0.01; *** p<0.001
